# Susceptibility and diffusion MRI biomarkers predict development of Parkinsonism in iRBD

**DOI:** 10.1038/s41531-025-01174-x

**Published:** 2025-11-21

**Authors:** Zsoka Varga, Jiri Nepozitek, Jan Hlavnicka, Jiri Keller, Abhineet Ojha, Patrizia Pantano, Stanislav Marecek, Karel Sonka, Petr Dusek

**Affiliations:** 1 Department of Neurology and Center of Clinical Neuroscience, First Faculty of Medicine, Charles University and General University Hospital in Prague, Prague, Czech Republic; 2 Department of Circuit Theory, Faculty of Electrical Engineering, Czech Technical University in Prague, Prague, Czech Republic; 3 Radiodiagnostic Department, Na Homolce Hospital, Prague, Czech Republic; 4 Third Faculty of Medicine, Charles University in Prague, Prague, Czech Republic; 5 Department of Human Neurosciences, Sapienza University of Rome, Rome, Italy; 6 IRCCS Neuromed, Pozzilli, IS Italy

**Keywords:** Neurological disorders, Movement disorders, Parkinson's disease

## Abstract

Quantitative MRI techniques, including quantitative susceptibility mapping (QSM) and diffusion tensor imaging (DTI), may detect early neurodegenerative changes in ɑ-synucleinopathies, especially within the midbrain. This study evaluated their potential to predict phenoconversion to overt synucleinopathy in 79 patients with isolated REM sleep behavior disorder (iRBD) followed annually over 5.6 ± 3.0 years. Phenoconversion, defined by emergence of parkinsonism or dementia, occurred in 21 patients. Baseline QSM and DTI data were analyzed to identify regional brain differences, revealing increased magnetic susceptibility and fractional anisotropy (FA) in the bilateral cerebral peduncle of phenoconverters. Increased magnetic susceptibility and FA within this region were associated with higher phenoconversion risk (FA: hazard ratio (HR) = 1.84, susceptibility: HR = 1.67). Their combined score predicted phenoconversion with accuracy similar to dopamine-transporter imaging (HR 2.58 vs 2.85). Findings suggest that increased susceptibility and FA in the cerebral peduncle may serve as biomarkers of early phenoconversion, potentially reflecting compensatory neuroplastic changes in subthalamo-pallidal pathways.

## Introduction

Rapid eye movement (REM) sleep behavior disorder (RBD) is a parasomnia characterized by abnormal behavior during REM sleep that corresponds to dream content. If no primary conditions causing RBD are present, it is referred to as isolated RBD (iRBD), which is considered an early manifestation of neurodegenerative diseases associated with ɑ-synuclein aggregation, such as Parkinson’s disease (PD), dementia with Lewy bodies (DLB) and multiple system atrophy (MSA)^1–4^. Research on iRBD provides an opportunity to identify prodromal biomarkers that could track disease progression and predict the phenoconversion to overt ɑ-synucleinopathy^5^.

Over recent years, an increasing number of studies have explored neuroimaging markers that depict neurodegenerative brain changes in iRBD^6,7^. Among these, magnetic resonance imaging (MRI) techniques measuring iron concentration have been proposed as sensitive tools for detecting neurodegenerative changes^8^. Paramagnetic iron accumulation, visible as hypointense changes on susceptibility-weighted imaging (SWI), can be estimated from tissue magnetic susceptibility measured using quantitative susceptibility mapping (QSM)^9–11^. Increased brain iron content has been detected in PD and DLB^12–15^, typically, once motor or cognitive symptoms of ɑ-synuclein pathology are fully developed. More recent studies have also examined brain iron deposition in iRBD. The first study investigating brain iron content in iRBD using transverse relaxation rate (R2*) found no alterations^16^. Later studies identified abnormalities in the substantia nigra (SN) suggestive of iron deposits in iRBD using SWI^17^ and QSM^18^. Another QSM study found no significant difference in magnetic susceptibility between iRBD and healthy individuals, although a positive correlation between magnetic susceptibility in nigrosome 1 and disease duration was noted^19^. A recent voxel-based QSM study from our group documented increased magnetic susceptibility in the brainstem of iRBD patients, correlating positively with disease duration and abnormal muscle activity during REM sleep^20^. These findings suggest that magnetic susceptibility could serve as a progression biomarker in ɑ-synucleinopathies.

While QSM is mostly used to assess grey matter, local changes in magnetic susceptibility within white matter tracts are difficult to interpret due to their dependence on fiber orientation relative to the magnetic field^21^. However, diffusion tensor imaging (DTI) is commonly used to explore white matter abnormalities. Diffusion parameters such as fractional anisotropy (FA) and mean diffusivity (MD) are well-established markers of white matter tract integrity. DTI has been widely used to study white matter microstructure in PD, revealing heterogeneous pathological findings^22^. DTI has proven useful in distinguishing PD from healthy controls and monitoring its progression, as abnormalities correlate with the severity of motor and non-motor symptoms^23,24^. When it comes to iRBD, the findings also show a notable heterogeneity. Scherfer et al.^25^ found a significant decrease of FA in the tegmentum and periaqueductal gray matter and an increase of MD in the pontine reticular formation using a voxel-based approach. Another study by Unger et al.^26^ found an increase of FA in the internal capsule bilaterally and in the olfactory pathway using tract-based spatial statistics (TBSS). Pyatigorskaya et al.^27^ using the ROI-based approach found no significant difference in FA values; only a lateralized increase of MD in widespread locations was detected. Recently, a study by Holtbernd et al.^28^ found increased FA in the corticospinal tract and other non-motor tracts, the cerebellar peduncles and midbrain of iRBD patients compared to controls. Taking into account the heterogeneity of findings aside with diverse methodology used, it is hard to make any conclusions about DTI abnormalities in iRBD. Most of the described DTI changes are localized in the pontomesencephalic area or within surrounding tissues, similar to the studies employing imaging techniques based on magnetic susceptibility. This is in line with our understanding of the pathological basis of parkinsonism, assuming that dysfunction of the basal ganglia circuits underlies the clinical manifestation of akinesia and rigidity. Given these heterogeneous findings, no validated MRI marker could predict phenoconversion into manifest ɑ-synucleinopathy in iRBD. To date, the strongest and best-validated neuroimaging marker of phenoconversion into PD and DLB is considered dopamine transporter imaging^29,30^. On the other hand, high cost and limited availability of this technique can restrict its access, especially in underserved or resource-limited settings. Additionally, the use of ionizing radiation raises concerns about patient safety, particularly with repeated imaging.

This study aims to test whether quantitative MRI measures such as magnetic susceptibility and DTI parameters could be useful in predicting the risk of phenoconversion in iRBD patients.

## Results

### Participant characteristics

Altogether, 79 participants fulfilled the inclusion criteria. Their mean follow-up duration was 5.6 ± 3.0 years, ranging from 3 to 9 years. Of them, 21 (27%) converted to overt synucleinopathy, while 58 (73%) remained disease-free at the latest clinical follow-up exam. Twenty participants converted to PD, while a single participant converted to DLB, developing dementia first, followed by parkinsonism 3 years later. Given the low number of DLB converters, we could only analyze risk factors for the development of parkinsonism, and thus, we set the phenoconversion time at the onset of parkinsonism in the latter patient. The clinical and demographic characteristics of participants are summarized in Table 1. We observed no significant difference in demographic parameters between RBDpark and RBDnc at baseline, though the RBDpark group was, on average, two years older and included more men. There was also no significant difference in Unified Parkinson’s Disease Rating Scale part III (MDS-UPDRS-III), Montreal Cognitive Assessment (MoCA) score, and disease duration between the groups. Notably, the diagnoses of PD and dementia were based strictly on established clinical criteria and judgement of the examining clinician, rather than on scores from rating scales alone. Although several participants had relatively high MDS-UPDRS III scores or low MoCA scores, these findings can be attributed to our conservative scoring policy, which favors assigning higher scores in cases of uncertainty, and to factors such as low educational attainment or simplex personality in the case of cognitive assessment.

**Table 1:** Clinical and demographic data

	RBDpark *n* = 21	RBDnc *N* = 58	*P* value
Sex (male/female)	20/1	51/7	0.34
Age (years)	68.9 ± 5.1 [60–77]	66.4 ± 7.3 [46–79]	0.16
Disease duration at baseline (years)^a^	6.3 ± 4.7 [1–16]	7.2 ± 8.8 [1–59]	0.66
MDS–UPDRS-III score	7.4 ± 6.7 [2–24]	6.2 ± 5.2 [0–24]	0.39
MoCA score	23.6 ± 2.4 [19–27]	23.7 ± 3.2 [13–30]	0.92
UPSIT score	20.8 ± 8.7 [9–36]	22.9 ± 7.4 [6–37]	0.3
Mild cognitive impairment^b^ (%)	24%	24%	—

Values are reported as mean ± standard deviation; RBDpark – patients with isolated REM sleep behaviour disorder (iRBD) who developed parkinsonism; RBDnc – patients with iRBD that did not convert to manifest synucleinopathy.

*MDS-UPDRS* Movement Disorders Society-sponsored Revision of the Unified Parkinson’s Disease Rating Scale, *UPSIT* University of Pennsylvania Smell Identification Test, *MoCA* Montreal Cognitive Assessment.

^a^Disease duration is defined as the subjective duration of dream enactment behavior.

^b^Mild cognitive impairment defined as a MoCA score < 1.5 standard deviation below the age-related normative mean in the Czech population.

### Voxel-based analysis of MPRAGE, QSM, and DTI

Voxel-based morphometry on MPRAGE images in 79 participants showed no significant difference, neither on the whole brain nor on the upper mesencephalon level.

Of the 79 participants, two did not have the multi-echo GRE pulse sequence completed, nine were excluded from the QSM analysis during the quality control (not satisfying reconstruction or normalization), and another seven participants were excluded from the DTI analysis due to unsuccessful preprocessing. Therefore, 68 participants were eligible for the QSM analysis and 72 for the DTI analysis. Furthermore, since SN degeneration and neuroimaging abnormalities are expected to precede phenoconversion by only a limited period, we restricted the voxel-based QSM and DTI analyses to participants who converted within six years of the MRI examination (excluding two participants, who phenoconverted after 7 years, and two after 8 and 9 years, respectively), yielding 12/56 RBDpark/ RBDnc for the QSM analysis and 17/55 RBDpark/ RBDnc for the DTI analysis. A threshold was introduced based on an initial exploratory QSM analysis including all subjects, that showed no significant difference between the two groups. The six-year cutoff was selected based on extrapolated estimates of the earliest SN abnormalities from prior neuropathological^31^, positron-emission tomography^32^, and MRI^33^ studies.

**Fig. 1:**
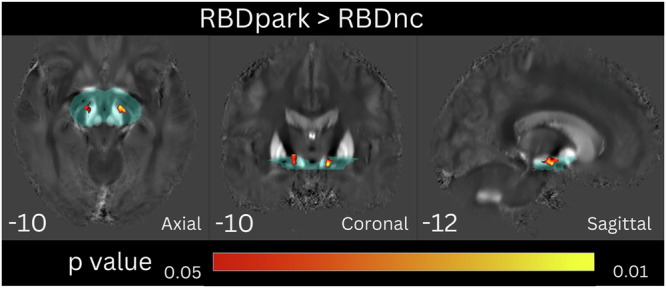
Voxel-wise QSM analysis on the upper mesencephalon level. QSM cluster-based between-group statistics using TFCE thresholded at FWE corrected *p* < 0.05, controlling for sex and age. Results are overlaid onto the mean study-wise QSM image in the MNI space, coordinates in mm are shown for each slice. The explicit hand-drawn upper mesencephalon mask is shown in transparent cyan color.

The voxel-based QSM analysis on the whole-brain level showed no significant differences between RBDpark and RBDnc. Using the upper mesencephalon mask, the RBDpark group had a significant symmetrical increase in susceptibility in the white matter area of the crura cerebri at the level of the SN and continuing cranially towards the striatum compared to the RBDnc group (Fig. 1). No significant differences were found with inverse contrast (i.e., RBDnc vs RBDpark).

The voxel-based analysis of the diffusion parameters did not show a significant difference in FA or MD between RBDpark and RBDnc on the whole brain, nor on the upper mesencephalon level. However, uncorrected results thresholded at *p* < 0.05 showed multiple small clusters with increased FA and decreased MD in the mesencephalon, including a region corresponding to the area of significant difference detected in the QSM analysis. Due to the low specificity of these uncorrected findings, we have chosen not to present these results.

### ROI analysis of QSM and DTI

Next, we retrieved quantitative FA, MD, and magnetic susceptibility values from the ROI defined by the significant difference in the voxel-based QSM analysis. The ROI-based analysis showed significant between-group differences with greater FA (*t* = −2.62, df = 70, *p* < 0.05) and greater magnetic susceptibility in the RBDpark group (*t* = −2.99, df = 66, *p* < 0.01; Table 2). None of the MRI markers were significantly associated with age in the RBDnc group. We also did not find any differences between men and women; however, the low statistical power suggests that this result does not provide strong evidence, especially for magnetic susceptibility (*t* = −1.9, df = 49, *p* = 0.06, post hoc power = 0.52).

**Table 2:** Summary of neuroimaging parameters and their statistical characteristics

	RBDpark	RBDnc	Group difference	~Age
Fractional anisotropy (−)	0.73 ± 0.03 (0.67, 0.78)	0.71 ± 0.03 (0.65, 0.78)	−0.67 * (−1.18, −0.15)	−0.19 N.S.
Magnetic susceptibility (ppb)	33 ± 21 (7, 88)	19 ± 16 ♀ (−12, 60)	−0.83 ** (−1.39, −0.26)	0.23 N.S.
Mean diffusivity (10^−6 ^mm²/s)	753 ± 49 (658, 835)	751 ± 38 (637, 833)	−0.04 N.S. (−0.55, 0.46)	0.06 N.S.
Mean putaminal DAT-SPECT z-score	−0.74 ± 1.22 (−2.47, 2.66)	0.56 ± 1.29 (−1.44, 5.00)	1.02 *** (0.49, 1.54)	0.00 N.S.
Putaminal DAT-SPECT z-score of the most affected hemisphere	−1.02 ± 1.24 (−2.67, 2.26)	0.39 ± 1.32 (−1.54, 4.90)	1.07 *** (0.55, 1.60)	−0.03 N.S.
Mean putaminal SBR	2.03 ± 0.48 (1.4, 3.31)	2.60 ± 0.53 (1.82, 4.38)	1.09 *** (0.56, 1.61)	−0.24 N.S. (*p* = 0.077)
Putaminal SBR of the most affected hemisphere	1.92 ± 0.49 (1.18, 3.14)	2.53 ± 0.54 (1.68, 4.37)	1.15 *** (0.61, 1.68)	−0.26 N.S. (*p* = 0.054)
LDA score (−)	0.58 ± 1.13 (−1.89-2.19)	−0.70 ± 1.33 (−3.12-2.19)	−0.99 *** (−1.57, −0.41)	−0.01 N.S.

Parameters of RBDpark and RBDnc groups are reported as mean ± standard deviation (minimal value - maximal value). All differences between groups were calculated via t-test and Wilcoxon rank sum test for normally and non-normally distributed data, respectively. Group differences in the 4th column are characterized by Cohen’s d with confidence intervals and p-value of the test. Cohen’s d was reported for all markers to make the results comparable, including the non-normally distributed putaminal DAT-SPECT z-score in the most affected hemisphere (*p* = 0.03) because of its moderate skewness (0.96). The effect of sex was tested only on RBDnc subjects to avoid possible bias of phenoconversion. The last column lists the age dependency of parameters in RBDnc group tested via Pearson’s correlation coefficient for normally distributed data and Spearman’s correlation coefficient for non-normally distributed data and its corresponding probability.

*ppb* parts per billion, *LDA* Linear Discriminant Analysis, *RBDnc* non-converting patients with REM sleep behavior disorder, *RBDpark* patients with REM sleep behavior disorder that converted to Parkinson’s disease during the study follow-up.

*p < 0.05, **p < 0.01, ***p < 0.001, N.S. = not significant.

♀*p* < 0.1, the effect of sex was not significant, but the post hoc power of the test was lower than 0.8 due to the small representation of females.

The comparison with clinical parameters showed a significant correlation between the MDS-UPDRS III score and magnetic susceptibility (*R* = 0.25, *p* = 0.037) and MD (*R* = 0.24, *p* = 0.044) within the ROI. There was no significant correlation between any MRI metrics and the MoCA score. There was also no significant correlation between diffusion parameters and magnetic susceptibility values within the ROI.

### Comparison of MRI and DAT-SPECT parameters

The proportion of patients with abnormal dopamine transporter single-photon emission computed tomography (DAT-SPECT) was 52% in the RBDpark and 11% in the RBDnc group (*p* < 0.001). Compared to RBDnc, the RBDpark group had significantly lower mean DAT-SPECT putaminal z-score (*t* = 4.01, df = 74, *p* < 0.001), as well as DAT-SPECT putaminal z-score in the most affected hemisphere (*z* = 4.17, *p* < 0.001; Table 2). DAT-SPECT z-score of the putamen in the most affected hemisphere showed an area under the curve (AUC) of 81% for discriminating RBDpark and RBDnc. The cut-off −1 z-score showed an accuracy of 79%, sensitivity of 53%, and specificity of 89% for identifying RBDpark. The optimal cut-off for our dataset, determined by the Youden Index was estimated to be −0.63 with an accuracy of 77%, sensitivity of 76%, and specificity of 77%. Magnetic susceptibility (AUC = 70%, *D* = 1.39, *p* = 0.08) and FA (AUC = 68%, *D* = 1.32, *p* = 0.09) had comparable ROC with DAT-SPECT z-score of the putamen in the most affected hemisphere, while MD (AUC = 51%, *D* = 2.79, *p* < 0.01) performed significantly worse.

From MRI parameters, only magnetic susceptibility was significantly correlated with the mean DAT-SPECT z-score of the putamen (*R* = −0.38, *p* < 0.01) and mean putaminal specific binding ratios (SBR) from both hemispheres (*R* = −0.43, *p* < 0.001).

### Classification experiment based on QSM and DTI

**Fig. 2:**
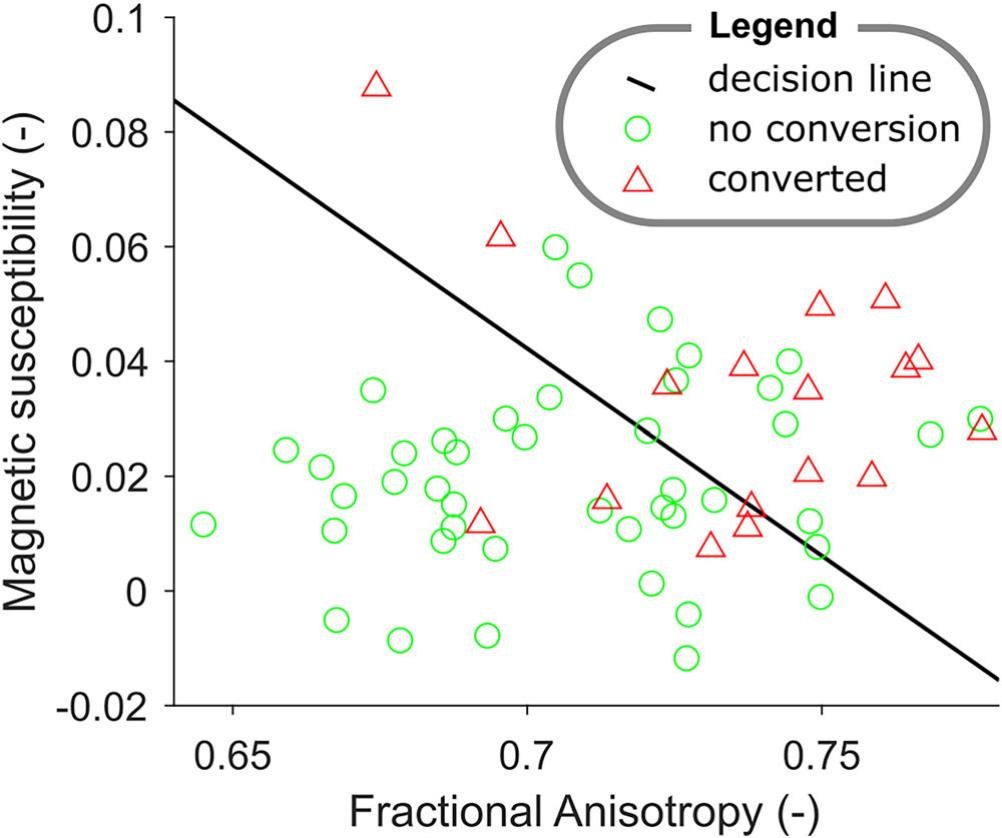
Linear discriminant analysis results. Illustration of the measured characteristics for participants who converted (red triangles) and participants who did not convert (green circles). The decision boundary is depicted as a black line and represents a model estimated on the whole data without cross-validation.

The classification experiment was conducted in 61 iRBD patients (17 subjects converted) with all (QSM and DTI) data available. LDA score was significantly different in RBDpark and RBDnc (*p* < 0.001; Table 2). The LDA classifier showed an accuracy of 70%, sensitivity of 71%, specificity of 70%, and AUC of 76%. The ROC performance was comparable with the DAT-SPECT z-score of the putamen in the most affected hemisphere (*D* = 0.54, *p* = 0.29). Please see Fig. 2 depicting all observations and the final decision boundary. Please see Supplementary Figure S1 illustrating the receiver operating characteristics of the classifier as well as individual MRI biomarkers.

### Phenoconversion hazard analyses

All parameters were normalized to z-scores before Cox regression based on their characteristics in Table 2. Reported HRs of DAT-SPECT parameters represent results with reversed signs, so an increased value means a worse effect of the disease for all reported markers. All cut-offs used in Kaplan-Meier plots were applied to the original raw values of markers.

**Fig. 3:**
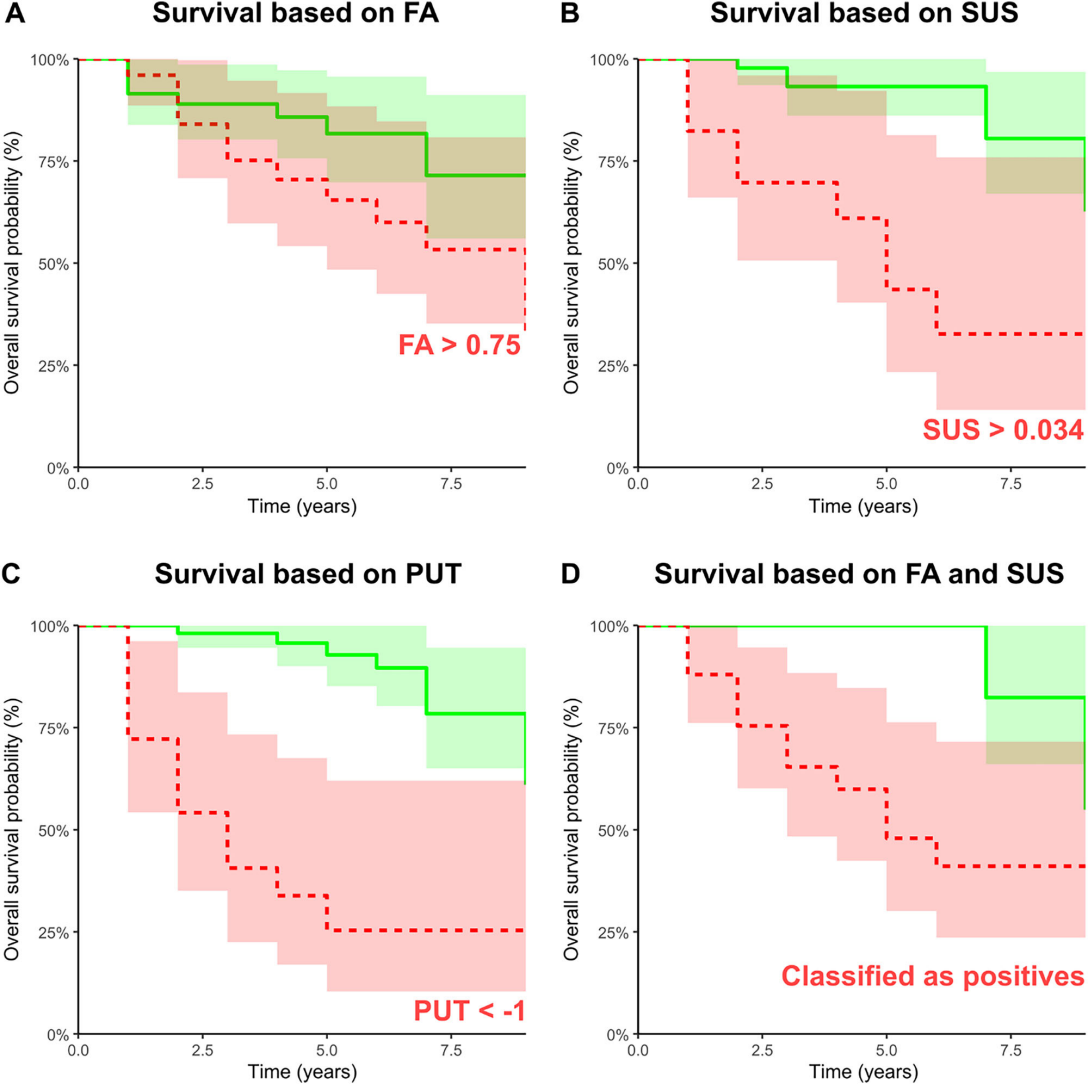
Survival analyses. Kaplan-Maier plots for FA (**A**), SUS (**B**), PUT (**C**), and a combination of FA and SUS (**D**) determined in the classification experiment. Each subplot contains a green line representing subjects identified as negative and a red line representing subjects identified as positive based on testing the marker with the optimal cut-off value listed in each figure. The categorization in subplot D was based on the classifier’s final decision utilizing two-dimensional data from FA and SUS. The confidence intervals are plotted as areas. Note that results are presented within subsets from which missing values were removed. FA = fractional anisotropy of MRI, SUS = magnetic susceptibility, PUT = DAT-SPECT z-score of putamen of the most affected hemisphere.

A total of 16 men and 1 woman of 68 subjects with available QSM data phenoconverted, with a mean age of 69.0 ± SD 5.2 years and a mean follow-up duration of 4.4 ± 2.8 years. After adjusting for age (HR = 1.06; 95% CI = 0.98, 1.15; *p* = 0.13), the z-score of magnetic susceptibility was positively associated with conversion (HR = 1.67; 95% CI = 1.16, 2.39; *p* < 0.01); see the Kaplan-Meier plots in Fig. 3A.

A total of 20 men and 1 woman of 72 subjects with available FA data phenoconverted, with a mean age of 68.9 ± SD 5.1 years and a mean follow-up duration of 3.9 ± SD 2.7 years. The z-score of FA was positively associated with conversion (HR = 1.84; 95% CI = 1.10, 3.10; *p* < 0.05) when controlling for age (HR = 1.11; 95% CI = 1.03, 1.20; *p* < 0.01); see the Kaplan-Maier plots in Fig. 3B.

A total of 20 men and 1 woman out of 76 subjects with available DAT-SPECT data phenoconverted. The mean age at phenoconversion was 68.9 ± SD 5.1 years, with a mean follow-up duration of 3.9 ± SD 2.7 years. DAT-SPECT putaminal z-score in the most affected hemisphere was negatively associated with phenoconversion (HR = 2.85; 95% CI = 1.73, 4.70; *p* < 0.001), after adjusting for age (HR = 1.04; 95% CI = 0.98, 1.12; *p* = 0.22). Refer to the Kaplan-Meier plots in Fig. 3C for further details.

Score determined by the linear discriminant analysis of magnetic susceptibility and FA was found to be significantly associated with phenoconversion (HR = 2.58; 95% CI = 1.47, 4.54; *p* < 0.001), after adjusting for age (HR = 1.10; 95% CI = 1.01, 1.19; *p* < 0.05). Refer to the Kaplan-Meier plots in Fig. 3D for further details.

### Laterality of MRI biomarkers

Average FA in the right hemisphere (0.7187 ± SD 0.0319) was not significantly different from the left hemisphere (0.7157 ± SD 0.0405) (Student’s *t* test, *p* = 0.62). Average measures of magnetic susceptibility in the right hemisphere (15.25 ± 20.00 SD) were significantly smaller than in the left hemisphere (27.83 ± 21.00 SD) (Student’s *t* test, *p* < 0.001). Average MD in the right hemisphere (752.85 ± 43.00 SD) was similar to left hemisphere (751.04 ± 58.00 SD) (Student’s *t* test, *p* = 0.83).

To compare the laterality of MRI biomarkers with DAT-SPECT, we defined laterality measures as the difference between left and right hemispheres for susceptibility, FA, MD, and DAT-SPECT of the putamen. We observed no correlation between laterality of susceptibility and DAT-SPECT of the putamen (*R* = 0.09, *p* = 0.48), FA and DAT-SPECT of the putamen (*R* = −0.09, *p* = 0.45), or MD and DAT-SPECT of the putamen (*R* = −0.01, *p* = 0.91). (Supplementary Fig. 1.).

To further test whether laterality in MRI biomarkers requires special consideration, we compared the AUC of MRI biomarkers calculated over both hemispheres versus the AUC of the worst-performing hemisphere for each biomarker. Overall FA showed an AUC of 0.68, which was not substantially different from worst-hemisphere FA with an AUC of 0.69. Overall susceptibility showed an AUC of 0.70, which was not substantially different from worst-hemisphere susceptibility with an AUC of 0.65. AUC of overall MD of 0.51 was slightly better than AUC of the worst-hemisphere MD with an AUC of 0.42.

We acknowledge that the dataset may be insufficient to draw definitive conclusions from these analyses. The observed hemispheric differences in susceptibility measurements could potentially be attributed to asymmetric region-of-interest characteristics, where one hemisphere exhibits a slightly larger coverage area while the contralateral side shows a more elongated configuration. This asymmetry may result in the inclusion of anatomically distinct regions with inherently different baseline susceptibility values, thereby confounding the comparative analysis between hemispheres.

Therefore, we conclude that incorporating laterality considerations into our MRI measurement analysis design would not be beneficial for this study.

## Discussion

This is the first study examining QSM and DTI changes in iRBD patients in relation to phenoconversion. We have shown that abnormal magnetic susceptibility and FA values in a region located in the crura cerebri predict phenoconversion with an accuracy comparable to DAT-SPECT^34^. Of the patients who phenoconverted, the majority developed PD and the detected MRI changes were localized to the mesencephalic region, therefore we can only draw conclusions regarding the development of parkinsonism. The marked predominance of PD converters (95%) is somewhat atypical for iRBD studies, in which the usual distribution of phenoconversion to PD is around 50%^35,36^. This uneven distribution could be attributable to the relatively small sample size; however, we cannot exclude the possibility of a population-specific effect in the Czech cohort, similar to what has been observed, e.g. in Asian patients, where MSA conversions are more frequent^37^.

While QSM is typically applied to gray matter due to its sensitivity to iron content, it also captures susceptibility changes in adjacent white matter regions, particularly those rich in myelin or iron^38,39^. This means, susceptibility differences found outside the SN may still reflect disease-relevant changes, highlighting the broader applicability of QSM beyond gray matter alone.

Several previous works described elevated magnetic susceptibility values in the mesencephalic area of PD patients, even in the early stage^40,41^. Similarly, increased magnetic susceptibility in the mesencephalon, mostly in the SN has been described in PD and iRBD patients^17–20,42–45^. Surprisingly, our study did not find a clear increase in magnetic susceptibility values within the SN, but rather in the adjacent white matter. This contrasts with our previous study^42^, which used a nearly identical iRBD cohort and demonstrated significantly elevated susceptibility in the SN of iRBD patients when compared to controls, although no correlation with motor or cognitive scores was observed. As most of the patients in the previous study had not yet phenoconverted, we assume that these susceptibility changes may reflect ongoing neurodegenerative processes in the SN that are not necessarily directly linked to early phenoconversion or the severity of motor and cognitive symptoms. Most of previous studies used an explicit mask of the SN or nigrosomes, excluding the surrounding white matter tracts from the analysis. We assume that the mesencephalic white matter changes extending towards the striatum, as observed in our study, may only arise relatively shortly before phenoconversion. Consequently, these alterations could be overlooked in group studies that include iRBD patients at various stages of progression.

DTI has been widely used in studying PD white matter microstructure abnormalities. A recent meta-analysis^22^ on diffusion changes in PD based on 39 published articles reported a significant FA increase in the basal ganglia, midbrain, and corticospinal tract in early-stage PD patients. This finding strongly supports our result where the increase of FA in the midbrain is detectable in iRBD patients bound for phenoconversion into PD.

Regarding previous findings of DTI abnormalities in iRBD, our study supports the findings of Unger et al.^26^, which presented a similarly increased FA in the bilateral internal capsule. In the study by Pyatigorskaya et al.^27^, we believe that using the strict SN mask could have prevented detecting changes outside this region. The composition of the patient cohort could explain the negative findings of Ohlhauser et al.^46^, as they used patients with iRBD mixed with patients with hyposmia.

Spatially overlapping abnormal findings in two independent quantitative MRI metrics in our current study are highly indicative of the existence of a common pathogenic process in this location. Moreover, the correlation with subthreshold motor symptoms of parkinsonism and, in the case of susceptibility, also with the degree of nigrostriatal dopaminergic denervation indicates a strong connection with the substantia nigra pathology despite the region being located in white matter.

Both alterations in white matter (of FA and magnetic susceptibility), may reflect microstructural changes likely related to the arrangements of axonal fibers. At places with multiple crossing fibers an increase of FA may not only signal an increased organization of white matter tracts, but also a selective degeneration of fibers, oriented in other than dominant direction. Indeed, there are multiple crossing fibers in the region with significant changes, involving the corticospinal tract and fibers interconnecting the globus pallidus and the subthalamic nucleus^47^. The region found in our study corresponds with the subthalamic-pallidal and pallido-subthalamic fibers reported by Pujol et al.^47^. Interpreting changes in magnetic susceptibility in white matter is difficult. It may be associated with altered orientation of axonal fibers to the magnetic field, decreased myelin content or increased iron in the tissue. In the white matter, this can be a result of iron accumulation in activated microglia^48^ as part of the neurodegenerative process, spreading from the mesencephalic area gradually towards the striatum, causing the gradual loss of dopaminergic neurons and leading to the manifestation of clinical symptoms of parkinsonism. Our findings also suggest that QSM may be more sensitive than DTI in detecting white matter pathology in PD. There is only one study comparing diffusion parameters and magnetic susceptibility in the white matter of PD patients^39^, that found overlapping white matter changes on QSM and DTI imaging, also correlating with motor symptoms. These regions, however, were different from those found in our study and there were no conclusions about which MRI technique was more sensitive. We speculate that iron accumulation in the tissue, detected by QSM, could be a less specific but more sensitive sign of underlying pathology than alteration of axonal fibers. However, due to the challenges in interpreting magnetic susceptibility changes in white matter, QSM cannot be recommended as a sole method for comprehensive analysis of white matter abnormalities.

Our current understanding of PD pathology suggests that the occurrence of clinical symptoms is delayed by compensatory mechanisms aimed at mitigating the effect of neurodegeneration and preserving basal ganglia function for as long as possible. As pathological ɑ-synuclein damages nigral dopaminergic neurons, the surviving neurons undergo microstructural changes^49^ to maintain dopamine availability in the striatum. Additionally, the inhibitory external globus pallidus – subthalamic nucleus – internal globus pallidus (GPe-STN-GPi) network becomes hyperactive to reduce activity in the “direct” basal ganglia pathway^50^. Fan et al.^51^ demonstrated that the number of synaptic connections per GPe-STN axon terminal significantly predicts future dopaminergic loss. A recent review^52^ further suggests that these adaptive changes within the basal ganglia may occur in the early pre-symptomatic phase of the disease. These compensatory neuroplastic changes, namely the strengthening of the fibers of the GPe-STN-GPi network could provide a direct and straightforward explanation of the elevation of FA in this location. The fact that the increasing FA is also affecting the risk of phenoconversion can be explained by the fact that compensatory mechanisms progressively increase with more advanced neurodegeneration.

Additionally, previous evidence indicates that elevated ferritin-bound iron concentrations in gray matter not only increase susceptibility but also influence diffusion parameters, such as increasing FA and decreasing MD^53,54^. Although these studies focused exclusively on gray matter, this effect could offer an alternative explanation for our findings. To conclude, without a neuropathological-MRI correlation study, the microstructural underpinnings of our MRI findings cannot be inferred.

Regarding hazard analysis and ROC, the predictive value of the DAT-SPECT z-score of the putamen in the most affected hemisphere was considerably better for identifying early conversion to synucleinopathy than MD but not better than FA and magnetic susceptibility. Although the combination of magnetic susceptibility with FA showed AUC (76%) relatively lower than DAT-SPECT (81%), the sensitivity of combined MRI markers was higher (71%) than DAT-SPECT (53%), which can be biased by the fact that we adopted a DAT-SPECT cut-off from another study. Note that the optimal DAT-SPECT cut-off for our data was better for all metrics. These results of MRI-based markers are remarkable when considering other factors such as radiation burden and cost of DAT-SPECT compared to faster and cheaper MRI. Our study thus represents the first step toward improving the diagnostics of early ɑ-synucleinopathy via more efficacious imaging techniques. Moreover, our results can improve our understanding of the physiological mechanisms of prodromal neurodegeneration.

The main limitation of this study is the relatively low number of phenoconverted subjects. A small number of converters can especially cause the hazard analysis to be unrepresentative. The low number of DLB subjects is closely related to this issue, and we can only interpret the findings in relation to parkinsonism. Another limitation of our clinical data lies in the observation of phenoconversion, which might not occur between two follow-ups but rather gradually over the course of several years. Yet, this limitation applies to the majority of phenoconversion studies in iRBD^55^. A predominance of male participants in iRBD studies, as observed here, may also represent a minor limitation, as it could affect the generalizability of the findings to female patients who may exhibit different clinical or neuroimaging profiles. Future studies should examine the observed abnormalities in the context of healthy controls, PD and DLB groups, which would allow for a more comprehensive analysis of ɑ-synucleinopathies.

This study points out the possible use of quantitative MRI methods like DTI and QSM in predicting phenoconversion in iRBD patients. Their combination may help to identify patients that have an increased risk of developing parkinsonism in the near future. This knowledge could be useful in clinical trials when selecting suitable candidates for testing neuroprotective treatments. Our findings also underline the importance of researching not only the neuronal tissue damage itself but also the compensatory mechanisms in the white matter occurring concurrently with the neurodegenerative process, which may contribute to the understanding of the complex pathophysiological process behind ɑ-synucleinopathies.

## Methods

### Research participants

The study was approved by the Ethics Committee of the General University Hospital in Prague, No. 11/15 and written informed consent was obtained from all participants. Patients were recruited either through referral to the sleep center of our clinic or via media advertisement, followed by a stepwise screening process consisting of an internet survey and a structured telephone interview with a sleep specialist^56^. The diagnosis of RBD was established according to the third edition of the International Classification of Sleep Disorders (ICSD-3)^57^, based on a history of dream-enacting behaviors and nocturnal video-polysomnography (vPSG) demonstrating excessive EMG activity during REM sleep. The exclusion criteria were as follows: age under 45 years, clinical signs of overt dementia or parkinsonism at baseline, RBD associated with narcolepsy, encephalitis, and head injury, antidepressant medication, or the presence of focal brainstem lesions on MRI indicative of secondary RBD.

### Clinical examination

All study participants underwent a comprehensive protocol consisting of medical history retrieval, neurological examination including the Movement Disorders Society-sponsored Revision of the MDS-UPDRS III^58^, olfactory assessment using the University of Pennsylvania Smell Identification Test (UPSIT)^59^ and neuropsychological examination including a clinical interview and the MoCA as a measure of global cognitive function^60^. The presence of mild cognitive impairment (MCI) at baseline was determined as a MoCA score lower than 1.5 standard deviation (SD) below the age-related normative mean in the Czech population^60^.

The participants were recruited gradually over a six-year period with annual follow-up visits, including a clinical neurological examination and a neuropsychological examination with MDS-UPDRS and MoCA scoring. Phenoconversion was recorded upon the presence of parkinsonism or dementia. Parkinsonism was diagnosed according to the MDS criteria^61^, which is the presence of bradykinesia in association with resting tremor and/or rigidity. Dementia was diagnosed according to the MDS criteria^62^. For the diagnosis of DLB, the consensual criteria were used^63^. The patients were divided into two subgroups, RBD-parkinsonism (RBDpark) and RBD-non-converter (RBDnc), based on phenoconversion status for the between-group analysis. We have chosen the term RBDpark for the converter subgroup, as 95% of our converters developed PD, and we were able to analyze only MRI risk factors associated with parkinsonism (see Results section).

### Dopamine transporter SPECT

Within one month of the initial MRI examination, DAT-SPECT was conducted using the tracer [123I]-2-b-carbomethoxy-3b-(4-iodophenyl)-N-(3-fluoropropyl) nortropane ([123I]FP-CIT, DaTscan®, GE Healthcare, Little Chalfont, Buckinghamshire, UK), following the European Association of Nuclear Medicine (EANM) procedure guidelines^64^. The full protocol is described in more detail elsewhere^65^. Automated semi-quantitative analysis was performed with DaTQUANT v 2.0 software (GE Healthcare, USA); SBR and their z-scores in both putamina were retrieved. For comparisons with MRI markers that were calculated over both hemispheres, we averaged SBR and z-scores of putamen in both hemispheres. For other analyses, we used values from the most affected hemisphere’s putamen. Abnormal DAT-SPECT was defined by −1 z-score in the putamen from the most affected hemisphere, according to Arnaldi et al.^30^.

### MRI examination and processing

MRI examination was performed on a 3T scanner (Siemens Skyra 3T, Siemens Healthcare, Erlangen, Germany) with a 32-channel head coil. MRI morphometry was based on axial 3D T1-weighted Magnetization Prepared Rapid Gradient Echo (MPRAGE, repetition time (TR) 2200 ms; echo time (TE) 2.4 ms; inversion time (TI) 900 ms; flip angle 8°; field of view (FOV) 230 × 197 × 176 mm; voxel resolution 1.0 × 1.0 × 1.0 mm^3^) pulse sequence. QSM was reconstructed from multi-echo gradient recalled echo (GRE; TR = 34 ms; six equispaced echoes between 4.9 and 29.5 ms; flip angle = 15°; FOV = 192 × 256 × 128 mm; voxel resolution = 0.57 × 0.57 × 2 mm^3^) pulse sequence. Diffusion tensor MRI was performed with TR = 10.5 s; TE = 93 ms; total 72 slices with voxel resolution 2 × 2 × 2 mm^3^; 30 noncolinear directions with b-value of 1000 s/m^2^ and one *b* = 0 s/m^2^ image.

GRE phase images from all 32 channels (each corresponding to a separate radiofrequency coil) were combined offline using the ASPIRE (A Simple Phase Image Reconstruction for multi-Echo data) method^66^. QSM was reconstructed from the brain-stripped GRE magnitude and phase images with the QSMbox software package (https://gitlab.com/acostaj/QSMbox) for MATLAB (MathWorks, Natick, Massachusetts, USA) using the previously validated multi-scale dipole inversion algorithm^67^. We used the default pipeline for coil-combined multi-gradient echo data, and the process involved re-slicing the complex GRE data to an isotropic (i.e., 0.57 × 0.57 × 0.57 mm^3^) voxel resolution via zero-padding.

MPRAGE images were co-registered to the first-echo GRE magnitude image and were thus spatially aligned with QSM images. A study-specific T1 template was created using the advanced normalization tools (ANTs) software (http://stnava.github.io/ANTs) by averaging the MPRAGE images of 15 randomly selected iRBD patients. A custom template was previously recommended for optimized VBM^68^. The MPRAGE images of all participants were normalized to the space of this template using the ANTs software. The deformation matrix obtained in this step was applied to the QSM images, bringing them to the template space. Normalized QSM images were smoothed using a Gaussian kernel with a full-width at half maximum of 2 mm before entering the voxel-wise group analysis. The small smoothing kernel was chosen to prevent the increase of the blurring bias of small structures. The results were brought to Montreal Neurological Institute (MNI) standard space using the normalize function in SMP12 (MATLAB).

Structural preprocessing was performed using FMRIB’s Software Library (FSL), version 6.0.5.1 (https://fsl.fmrib.ox.ac.uk/fsl)^69^. Diffusion data were visually inspected for artifacts and preprocessed using different tools from FDT (FMRIB Diffusion Toolbox, part of FSL). Images were corrected for eddy current distortion and head motion using a 12-parameter affine registration to each subject’s first no-diffusion weighted volume, and the gradient directions were rotated accordingly. Non-brain tissue was removed from the eddy-corrected images using the Brain Extraction Tool, creating a binary mask of the brain. Then, maps of FA and MD were estimated at the individual level using the DTIFIT tool by fitting a tensor model to the eddy-corrected and brain-masked diffusion data. Registration between diffusion, structural, and standard space images was performed within FDT. Transformation matrices and their inverses were created to transform images between native and MNI space.

### Voxel-based MRI analyses

Voxel-based analyses comparing the RBDpark and RBDnc groups were performed first on the whole-brain level and then on the upper mesencephalon level. We focused the analysis on the upper mesencephalon because the MRI changes associated with iRBD and PD were most consistently described in this region^40^. To this end, an explicit hand-drawn mask of the upper msesencephalon including the adjacent white matter tracts was created.

For voxel-based morphometry, T1w images were spatially normalized, segmented into grey and white matter, and smoothed with a 2 mm Gaussian kernel in SPM12. A general linear model (GLM) was used for the voxel-based comparison of the local white and gray matter density with total intracranial volume, age, and sex as covariates of no interest.

For between-group QSM voxel-wise analyses, spatially normalized QSM images were smoothed with a 2 mm Gaussian kernel in SPM12. According to previous studies, including our own, the results of voxel-wise QSM analyses are comparable for raw susceptibility values and values normalized to a reference region^42,70^. Thus, we have used raw QSM data for the analysis. For statistical comparison of morphometric and QSM data, non-parametric permutation analysis was performed using threshold-free cluster enhancement (TFCE) for SPM12 (http://www.neuro.uni-jena.de/tfce/) set to default parameters (*E* = 0.5, *H* = 2, number of permutations=5000), with FWE corrected p-value threshold level of 0.05. Age, sex, and total intracranial volume were used as covariates of no interest.

To investigate between-group differences in diffusion parameters, voxel-wise analysis of FA and MD images was performed using a standard GLM in FSL with age and sex as covariates. The statistical analysis was performed using TFCE and thresholded at FWE corrected p-value < 0.05.

### Region of interest (ROI) analysis

Based on QSM voxel-wise analysis, a region with significant differences between RBDpark and RBDnc groups was identified (see Results section). This area was binarized and brought to the MNI space using the normalize function in SPM12, and mean bulk magnetic susceptibility values within this ROI were extracted for each patient. Next, this ROI was applied to the normalized DTI images, and the FA and MD values were obtained using the *fslmeants* function in FSL. As there was no significant difference between magnetic susceptibility, FA, and MD values in the ROIs from the left and right side, we have chosen to use the mean values from both sides for further analysis. Hemispheric averaging simplifies the analysis and may reduce variability; however, it also carries the limitation of potentially masking subtle asymmetries, which are clinically relevant in the early, often unilateral, stages of PD. A detailed analysis justifying the bilateral averaging of MRI parameters is provided as a post-hoc analysis in the Results part: Laterality of MRI parameters.

### Statistical analysis

The normality of distributions was assessed using the Lilliefors test. Group comparisons were tested via t-test for normally distributed data and Wilcoxon’s rank-sum test for non-normally distributed variables. Proportions between groups were examined via the chi-square test. The minimum sample size to detect a large effect (Cohen’s d = 0.8) between RBDnc and RBDpark via t-test with ɑ of 0.05, power of 0.8, and group allocation ratio of 0.28 was estimated to be 58 RBDnc and 16 RBDpark^71^. The relationships between clinical variables were analyzed via Pearson’s correlation coefficient for normally distributed data and Spearman’s correlation coefficient for non-normally distributed data. Between-group differences were calculated using ANCOVA with age and sex as covariates using Python in a Jupyter-notebook environment. The threshold of significant probability was set to *p* < 0.05.

We calculated the classification performance of individual DAT-SPECT and MRI parameters. We compared the ROC curves via a bootstrap test with 2000 iterations and an alternative single-sided hypothesis that DAT-SPECT parameters are better predictors of phenoconversion^72^. Additionally, we evaluated a combination of both FA and magnetic susceptibility in a classification experiment utilizing Linear Discriminant Analysis (LDA) within a leave-one-out cross-validation scheme. In addition to the classification outcome, we determined an LDA score as the ratio of the posterior probabilities on the logarithmic scale. Only subjects with all available data were included. The classification performance was quantified with sensitivity, specificity, accuracy, and AUC. The classification experiment was implemented in MATLAB and R language.

We performed a survival analysis of FA, magnetic susceptibility, and DAT-SPECT putaminal z-score from the most affected hemisphere via Cox proportional hazards regression to test the relationship between experiment duration and measured variables while accounting for age as a covariate. Nonlinearity was tested by inspecting the deviance of Martingale residuals. Variables that did not pass the assumptions tested with the weighted residuals test^73^ were modeled with the ridge regression with penalty calculated based on rescaling the predictors to have unit variance^74^. Magnetic susceptibility, FA, and LDA’s score were normalized to z-score to improve the numerical stability of the fitting procedure and comparability of results. Normalization utilized only the RBDnc group to avoid possible bias due to phenoconversion. Age-dependent markers were normalized by fitting a regression line and analyzing its residuals. The sign of DAT-SPECT z-scores was inverted for the Cox analysis to ensure similar interpretability of HR with other metrics. Survival analysis was conducted in R language with the Survival library available in CRAN^75^. We assume 10 minimum events per variable^75^. We did not control the family-wise error rate as each feature is related to an independent hypothesis. Missing values were removed from the statistical analysis.

## Supplementary information

Supplementary figure

## Data Availability

The datasets used and analyzed during the current study are available from the corresponding authors on request.
