# Supplementary material for: Susceptibility and diffusion MRI biomarkers predict development of Parkinsonism in iRBD
Source: NPJ Parkinsons Dis. 2025 Nov 21;11:332. doi: 10.1038/s41531-025-01174-x (PMC12639090; doi:10.1038/s41531-025-01174-x)
Supplement: Supplementary file 1 — Supplementary figure [file 41531_2025_1174_MOESM1_ESM.docx]

# Supplementary figure 1. Receiver operating characteristics

| 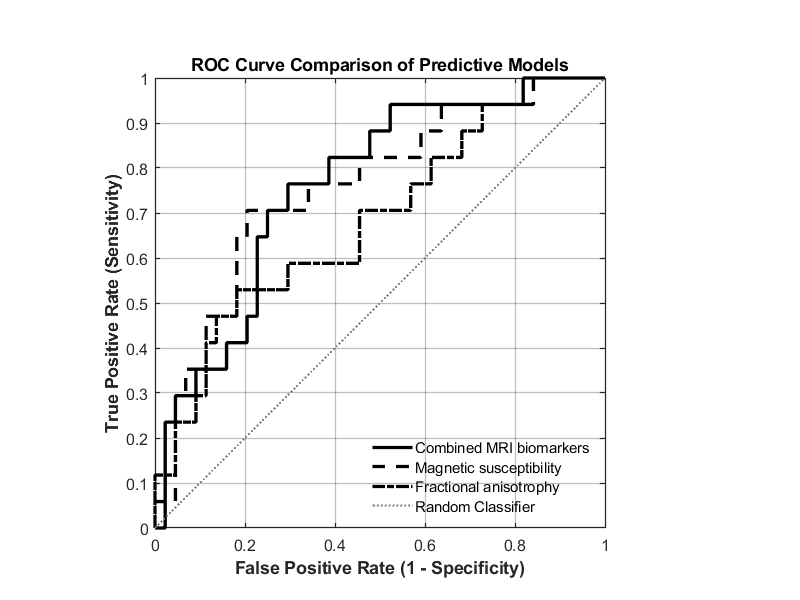 |
| --- |
| Figure S1: Receiver Operating Characteristic (ROC) curves comparing the predictive performance of three models for conversion prediction. The logistic regression model of combined MRI biomarkers (solid line), fractional anisotropy (dashed line), and magnetic susceptibility (dash-dot line) are juxtaposed to the diagonal reference line representing random classification. |
